# Computed tomographic features of pulmonary lesions in periarticular versus systemic histiocytic sarcomas in dogs

**DOI:** 10.3389/fvets.2026.1917708

**Published:** 2026-08-17

**Authors:** Moritz Folie, Ahmed Abdellatif, Julia Gedon, Jan Wennemuth

**Affiliations:** Small Animal Hospital Hofheim IVC Evidensia, Hofheim am Taunus, Hesse, Germany

**Keywords:** dendritic cell tumors, histiocytic sarcoma/diagnostic imaging, lung distribution pattern, pulmonary nodules, thoracic imaging

## Abstract

**Introduction:**

The aim of this retrospective study was to characterize and compare the computed tomographic (CT) appearance of lung lesions in periarticular histiocytic sarcomas (PAHS) and systemic histiocytic sarcomas (HS), identifying potential radiological differences.

**Methods:**

The medical records of 45 dogs diagnosed with PAHS or systemic HS, undergoing thoracic CT imaging were reviewed. CT images were evaluated for various parameters.

**Results:**

Results revealed that pulmonary lesions associated with systemic HS had larger lesion volumes, were mainly located ventrally 20/26 (77%) and were bronchocentric 23/26 (88%), often with irregular shapes 20/26 (77%) and air bronchograms 26/26 (100%). In contrast, PAHS pulmonary lesions were located predominantly interstitial 12/19 (63%), appearing round to ovoid 15/19 (79%) with less commonly observed air bronchograms 7/19 (37%). In systemic HS, lesion distribution showed no correlation with lung lobe volume (Spearman’s *ρ* = 0.14; *p* = 0.78717), with lesions most frequently observed in the right middle lung lobe 16/26 (62%). Conversely, in patients with PAHS, the distribution of lesions within the lung parenchyma showed a strong correlation with the lung lobe volumes (Spearman’s *ρ* = 0.94; *p* = 0.0048). Intrathoracic lymph node involvement was more frequent in systemic HS 21/26 (81%) than PAHS 12/19 (63%), typically with larger volumes in systemic HS.

**Discussion:**

The nodular lung pattern in PAHS resembles the metastatic distribution seen in other high-grade sarcomas. Significant morphologic and distributional differences indicate distinct patterns of pulmonary involvement in PAHS and systemic HS, which may reflect differences in biological behavior or routes of metastatic spread and raise the possibility of distinct biological subtypes.

## 1 Introduction

Histiocytic sarcomas (HS) are malignant and highly aggressive neoplasms that are distinguished into three different forms depending on their cellular origin and on the clinical presentation of the disease (1, 2). The localized and disseminated HS originate from dendritic cells, whereas the haemophagocytic HS, a rare subtype of histiocytic sarcoma originates from macrophages (3). Morphologically and immunohistochemically the localized and disseminated HS are identical, with positive staining for CD18, Iba1 and CD11c and negative staining for CD11d (4, 5). CD11c and CD11d are detected on frozen tissue, which is rarely performed in veterinary practice. Classically, Iba1 and CD18 are used on paraffin-embedded tissue. It is currently impossible to determine the exact sublineages of dendritic cells from which histiocytic sarcomas arise as there are currently no definitive immunohistochemical markers available to distinguish the potential subtypes (6–8). However, they do differ due to their clinical manifestation.

The disseminated form is more common in Bernese Mountain Dogs and accounts for up to 25% of deaths in this breed, while the localized form is more prevalent in Flat-Coated retrievers (9, 10). HS is not limited to Bernese Mountain Dogs and Flat-Coated Retrievers; it has also been reported with some frequency in other breeds, including Rottweilers, Golden Retrievers and lately also Miniature Schnauzers and Pembroke Welsh Corgi Dogs (2, 9, 11–14).

Localized HS usually develops in one region, affecting the dermis and subcutaneous areas, tissues surrounding the joint, muscular tissue, and more rarely internal organs. Whereas the disseminated form of HS affects multiple sites, primarily including lungs, lymph nodes, liver, spleen and bone marrow (12, 14, 15).

The localized form involving tissues surrounding the joints is known as periarticular histiocytic sarcoma (PAHS) and typically develops in periarticular tissues of large appendicular joints, with the stifle, elbow, and shoulder being the most commonly affected (11, 16, 17). PAHS is the most common canine joint tumor (17). A prior joint disease increases the risk of developing a PAHS (16).

A previous study has demonstrated significant variability in the histomorphological features of HS between Bernese Mountain Dogs (BMDs) and Flat-Coated Retrievers (FCRs), suggesting different subtypes of HS (8). In that study, spindle cells were observed more frequently in FCRs than in BMDs, a characteristic possibly linked to the higher incidence of localized HS in FCRs (6, 8). This variation in histological presentation further supports the hypothesis that multiple subtypes of HS exist, potentially arising from different dendritic cell lineages or following different differentiation pathways. This is additionally supported by a detailed histologic and immunohistologic review of 180 Flat-Coated Retrievers bearing HS-like tumors, where a histiocytic–spindle–pleomorphic phenotype was mainly seen in limb tumors (18).

This retrospective study aimed to evaluate and describe the differences in the computed tomographic (CT) appearance of pulmonary lesions between PAHS and systemic HS. To our knowledge, the CT characteristics of lesions associated with the different forms have not yet been described and compared.

## 2 Materials and methods

### 2.1 Selection and description of subjects

This single-center retrospective study was conducted at the Small Animal Hospital Hofheim IVC Evidensia in Germany. All methods were carried out in accordance with the relevant guidelines and regulations. The data used in this study were part of routine clinical activity; therefore, no ethical committee approval was required. The hospital database was queried for dogs with a histopathologically or cytologically confirmed diagnosis of periarticular or systemic HS which received thoracic CT examinations as part of their work-up between January 2014 and December 2023. The diagnosis was established by cytology or histopathology of the periarticular lesion and/or the regional lymph node in most patients with PAHS, and of a pulmonary lesion in patients with systemic HS. Direct sampling of suspected metastases was not routinely performed due to small size and/or inaccessible location of the lesions. Consequently, pulmonary metastatic disease was diagnosed based on the confirmed diagnosis of histiocytic sarcoma together with the overall clinical and imaging findings.

Only dogs with pulmonary involvement affecting at least one lung lobe were included. Furthermore, dogs with systemic HS were required to have lesions in at least one additional extrapulmonary site (e.g., lymph nodes. liver, spleen, kidneys, or musculature) or multifocal pulmonary involvement consistent with disseminated disease. For inclusion in the PAHS group the primary lesion had to be identified in the periarticular tissue of a joint with regional lymphadenopathy and pulmonary involvement. Dogs with a single pulmonary lesion were included only if additional metastatic lesions were identified elsewhere, and the pulmonary lesion was considered most consistent with metastatic disease. Dogs with an isolated, unconfirmed pulmonary lesion without other evidence of metastatic spread were excluded. Patients with evidence of concurrent systemic or pulmonary disease unrelated to HS were also excluded from the study.

### 2.2 Data recording and analysis

The CT examinations were performed using either a Toshiba Activion 16 CT scanner or a Toshiba Aquilion RXL CT scanner (Toshiba Medical Systems, Japan). Both scanners were equipped with 16 detector rows. Patients were positioned in sternal recumbency under general anesthesia and images were acquired with a slice thickness of 1 mm. Pre- and postcontrast images were obtained in all but one patient, for whom only postcontrast images were available. Postcontrast images were acquired following intravenous administration of 2 mL/kg of either iobitridol (Xenetix 350, Guerbet, France) or iohexol (Accupaque 350, GE Healthcare, USA). CT images were reconstructed using soft tissue and lung algorithms.

The scans were reviewed by three radiologists, two Diplomates ECVDI (AA, JW) and a first year ECVDI Resident (MF) using a picture archiving and communication system (PACS) workstation (OsiriX, version 13.0.3 PixmeoViewer). The observers were aware of the diagnosis during the review. The images were evaluated for predefined features to ensure a consistent assessment. In cases of disagreement, a consensus was reached.

The images were evaluated for qualitative and quantitative features including the number and size of lesions, shape, lung lobe(s) affected, attenuation, localization within the parenchyma, distribution, the presence of intralesional cavitation, mineralization and air bronchograms. Additionally, presence of pleural effusion, regional and intrathoracic lymphadenopathy, lesions in other abdominal organs, and the primary site of the PAHS were noted.

The localization within the parenchyma was categorized as either “bronchocentric” or “interstitial.” A bronchocentric location was defined as lesions primarily centered around the bronchi and bronchioles characterized by an airway associated pattern. An interstitial distribution was defined as lesions involving the pulmonary interstitium, without a recognizable airway related distribution.

Only the largest pulmonary lesion was assessed for volume calculation, if more than one lesion was present. The formula for ellipsoid volumes was used to approximate the volume of the pulmonary lesions (Volume = 4/3 * *π* * length/2 * width/2 * height/2).

For the individual lung lobe volume in dogs measurements from the study by Rahn et al. were used (19).

Attenuation was measured in Hounsfield Units (HU) by placing an ROI in a representative area on pre- and post-contrast images of the lung lesions. Contrast enhancement was assessed subjectively by comparing pre- and postcontrast images and was categorized as mild, moderate, or marked. No predefined Hounsfield unit thresholds were used for this classification.

### 2.3 Statistics

Statistical analysis was performed using commercially available software (SPSS 29.0, IBM SPSS Statistics for Mac, Armonk, NY, USA). The datasets were tested for normality and homogeneity of variance. An unpaired *t*-test was used to compare parametric data, while the Mann–Whitney test was used for nonparametric data. The Chi-Square calculator was used to detect differences in the characteristics of the populations. Spearman’s rank correlation was used to assess the association between lung lobe volume and lesion distribution. The significance level was set at *p* < 0.05.

## 3 Results

A total of 45 dogs met the inclusion criteria. All dogs were diagnosed via cytology (34/45) or histopathology (11/45) of the periarticular lesion, regional lymph node, or pulmonary lesion.

Twenty-six dogs were diagnosed with systemic HS. In addition to pulmonary involvement, some patients also showed lesions in other organs such as liver, spleen, kidneys and musculature. The mean age at diagnosis was 7.8 ± 2.3 years, with a mean weight of 34.7 ± 11.8 kg. This group included nine intact males, four castrated males, four intact females, and nine spayed females. The most commonly affected breed was the Bernese Mountain Dog (17/26, 65%). Other affected breeds included two Miniature Schnauzers (2/26, 8%), three crossbreeds (3/26, 12%), one Flat-Coated Retriever (1/26, 4%), one Rottweiler (1/26, 4%), one Labrador Retriever (1/26, 4%), and one Golden Retriever (1/26, 4%).

Periarticular histiocytic sarcoma was diagnosed in 19 dogs. The mean age at diagnosis was 8.7 ± 1.9 years, with a mean weight of 33.7 ± 11.5 kg. This group consisted of three intact males, 10 castrated males, one intact female, and five spayed females. The dogs with PAHS presented with a larger variety of breeds; Flat-Coated Retrievers (4/19, 21%) and Bernese Mountain Dogs (3/19, 16%) were the most frequently affected. Other affected breeds included two Golden Retrievers (2/19, 11%), two Labrador Retrievers (2/19, 11%), two Australian Shepherds (2/19, 11%), one American Staffordshire Terrier (1/19, 5%), one Bullmastiff (1/19, 5%), one Cane Corso (1/19, 5%), one Shar Pei (1/19, 5%), and two crossbreeds (2/19, 11%).

The primary joints affected were the stifle in seven dogs (7/19, 37%), the shoulder in five dogs (5/19, 26%), and the elbow in five dogs (5/19, 26%). Two additional joints affected were the tarsus (1/19, 5%) and the temporomandibular joint (1/19, 5%).

There were no significant differences in age, sex or weight between the two groups.

### 3.1 Number of lung lesions

Lesion counts among patients diagnosed with systemic HS ranged from one to 18, with 20 out of 26 (77%) having three or fewer lesions. In contrast, patients in the PAHS group exhibited lesion counts ranging from one to over 50, with 11 out of 19 (58%) having more than three observed lesions. Notably, the PAHS group exhibited a higher median lesion count (4; IQR: 1.5–7; range 1 to >50) compared to the systemic HS group (3; IQR 1–3; range 1–18), though no statistically significant difference was found in the overall number of lesions between the two groups (*p* = 0.181).

### 3.2 Size of lesions

The median tumor volume of the largest lung lesion in the systemic HS group was significantly larger (*p* < 0.001) than that in the PAHS group, with median volumes of 103.7 cm^3^ (IQR: 33.1–155 cm^3^) in dogs with systemic HS and 2.5 cm^3^ (IQR: 0.5–7.5 cm^3^) in PAHS patients.

### 3.3 Shape

In the PAHS group, lung lesions were primarily round to ovoid, accounting for 15/19 (79%) cases. One patient (1/19, 5%) in this group presented with an irregular lesion, while three patients (3/19, 16%) exhibited both round to ovoid and irregular shapes. Conversely, the systemic HS group predominantly displayed irregular confluent-shaped nodules, observed in 20/26 (77%) of cases, with the remaining six patients (6/26, 23%) presenting a combination of irregular and round to ovoid lesions. The lesion shapes significantly differed between the two groups (*p* < 0.001).

### 3.4 Distribution

In systemic HS, pulmonary lesions were mainly located ventrally (20/26, 77%). One patient (1/26, 4%) had a lesion located dorsally, while in the remaining five patients the nodules were distributed without a clear pattern (5/26, 19%).

In contrast, in the PAHS group, lesions were distributed throughout the lungs (11/19, 58%). Ventral lesions were present in four cases (4/19, 21%), while two patients (2/19, 11%) exhibited dorsal lesions and another two (2/19, 11%) had lesions in the mid-lung region.

The differences in lesion distribution between periarticular and systemic histiocytic sarcoma were significant (*p* < 0.001).

In addition, there was a strong correlation between lesion distribution and lung lobe volume in the PAHS group (Spearman’s *ρ* = 0.94; *p* = 0.0048). Lung lobes with larger volumes showed a higher number of lesions. In systemic HS, the lesion distribution showed no correlation with the lung lobe volume (Spearman’s *ρ* = 0.14; *p* = 0.78717). However, the most common location was the right middle lung lobe, which was involved in 16/26 (62%) of the patients.

### 3.5 Localization within the parenchyma

In the PAHS group, lesions were predominantly located interstitially in 12/19 cases (63%). One patient showed a bronchocentric location (5%), while 6/19 dogs (32%) exhibited both interstitial and bronchocentric nodules. In contrast, systemic HS lesions were primarily bronchocentric, observed in 23/26 cases (88%), with 3/26 dogs (12%) showing a combination of interstitial and bronchocentric lesions. The location of the lesions within the lung parenchyma differed significantly between systemic HS and PAHS (*p* < 0.001).

### 3.6 Attenuation

The mean pre-contrast attenuation was 37 ± 11 HU for PAHS and 43 ± 6.2 HU for systemic HS. Both groups showed mild to moderate contrast enhancement with a mean increase in attenuation of 32 ± 13.2 HU in PAHS and 29.5 ± 12 HU in systemic HS. The mean post-contrast attenuation was 67.5 ± 16.7 HU for PAHS and 71 ± 13.3 HU for systemic HS, with no significant difference in contrast enhancement observed between the groups (*p* = 0.461).

### 3.7 Presence of air bronchograms

An air bronchogram was observed in at least one lesion in each patient of the systemic HS group (26/26, 100%), which significantly differed (*p* < 0.001) from the PAHS group, where air bronchograms were less common (7/19, 37%).

**Figure 1:**
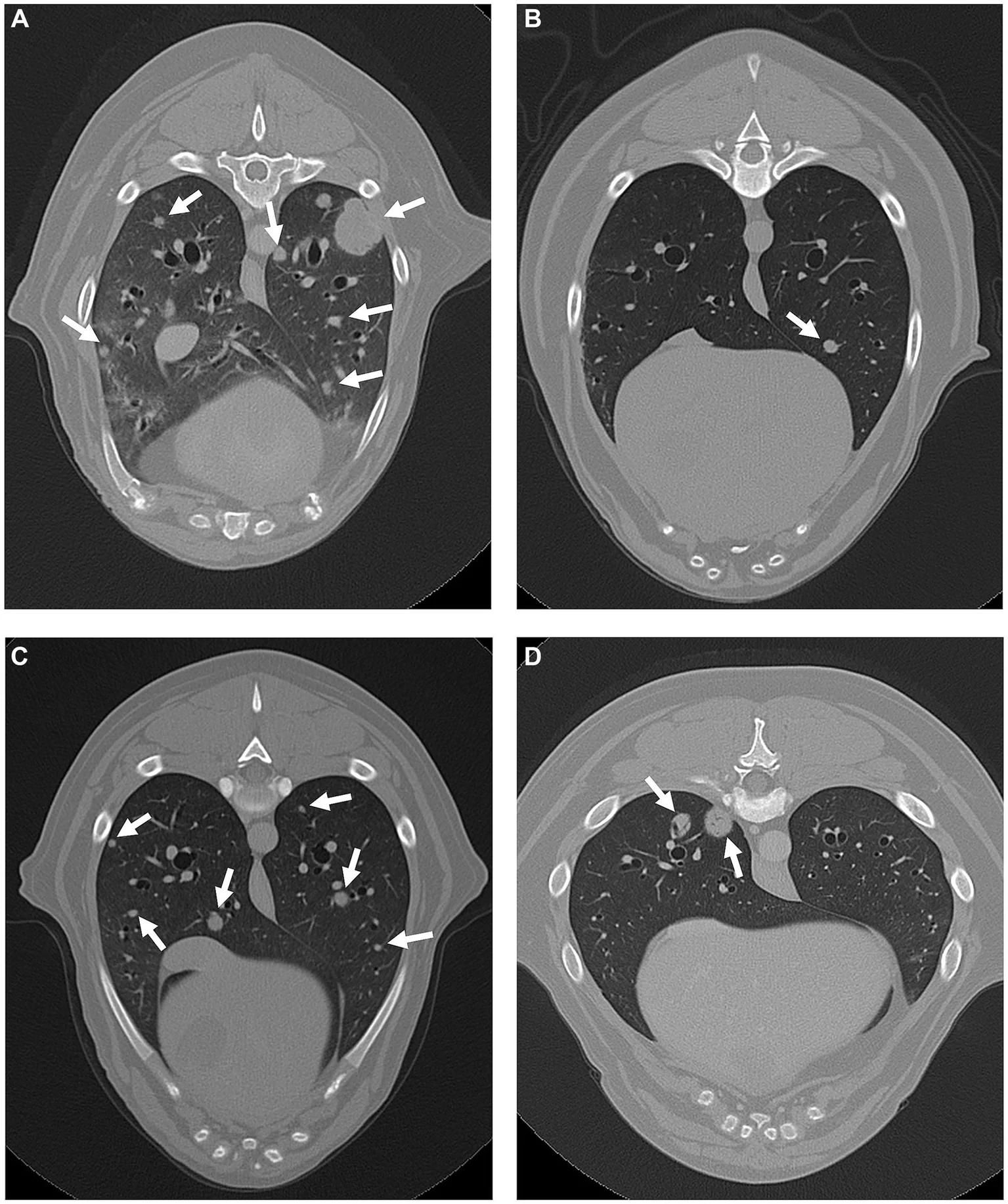
Transverse CT images of different patients included in the periarticular histiocytic sarcoma (PAHS) group displayed with a lung reconstruction algorithm (WL = −100, WW = 2,500). The patient’s right is to the left of the image. **(A)** Crossbreed, 8-year-old, spayed female, primary lesion—left elbow: Cross-sectional view at the level of the caudal aspect of the heart. Multiple round to ovoid soft tissue nodules (a couple of them marked with arrows) in different sizes and generally distributed in the lung parenchyma of the left and right caudal lung lobe. **(B)** Flat-Coated Retriever, 7-year-old, castrated male, primary lesion—left elbow: Cross-sectional view at the level of the cranial aspect of the liver. One round soft tissue nodule (arrow) is visible in the left caudal lung lobe. **(C)** Australian Shepherd, 8-year-old, male, primary lesion—left stifle: Cross-sectional view at the level of the cranial aspect of the liver. Multiple round to ovoid interstitial soft tissue nodules (a couple of them marked with arrows) in different sizes and generally distributed in the lung parenchyma of the right accessory and the left and right caudal lung lobe. **(D)** Bullmastiff, 6-year-old, spayed female, primary lesion—right stifle: Cross-sectional view at the level of the cranial aspect of the liver. Two round soft tissue nodules (arrows) are displayed in the right caudal lung lobe. In both nodules air bronchograms are clearly visible.

**Figure 2:**
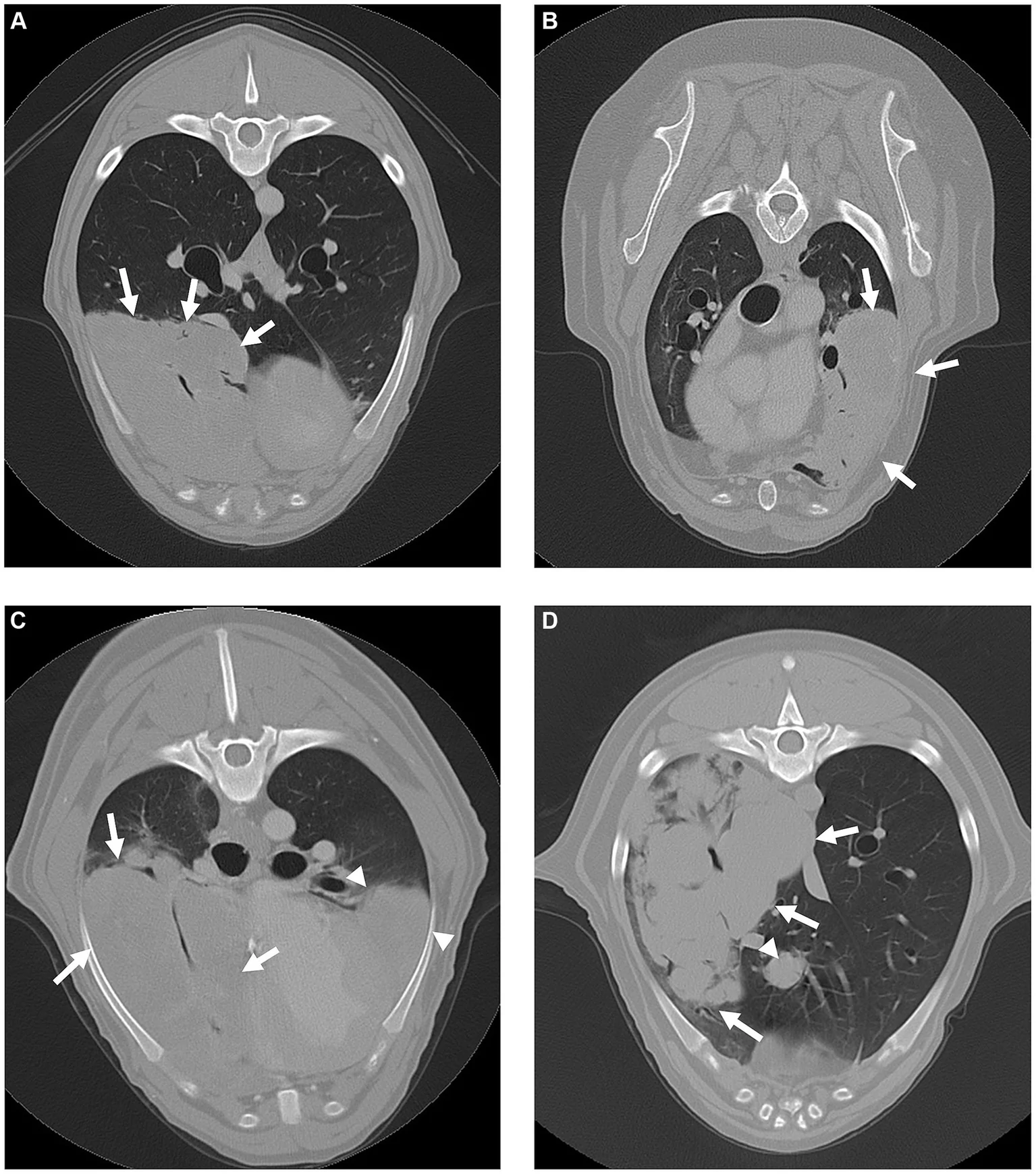
Transverse CT images of different patients included in the systemic histiocytic sarcoma (HS) group displayed with a lung reconstruction algorithm (WL = −100, WW = 2,500). The patient’s right is to the left of the image. **(A)** Bernese Mountain Dog (BMD), 7-year-old, male: Cross-sectional view at the level of the caudal aspect of the heart. A large irregular soft tissue lesion with a bronchocentric location and central air bronchograms is clearly visible in the ventral aspect of the right middle lung lobe (arrows). **(B)** BMD, 5-year-old, male: Cross-sectional view at the level of the cranial aspect of the heart. A large irregular soft tissue pulmonary lesion with a bronchocentric location and air bronchograms is shown in the ventral aspect of the left cranial lung lobe (arrows). **(C)** BMD, 4-year-old, male: Cross-sectional view at the level of the middle aspect of the heart of the patient. A large irregular soft tissue pulmonary lesion with a bronchocentric location and air bronchograms is clearly visible in the ventral aspect of the right middle lung lobe (arrows). In addition, in the caudoventral aspect of the left cranial lung lobe a second large lung lesion (arrowheads) is detectable. **(D)** BMD, 4-year-old, spayed female: Cross-sectional view at the level of the caudal aspect of the lung. A large irregular soft tissue lesion with a bronchocentric location and air bronchograms is displayed dorsally in the right caudal lung lobe (arrows). An additional smaller nodule (arrowhead) is present in the middle aspect of the right accessory lung lobe.

These morphologic and distributional characteristics of pulmonary lesions are shown in Figure 1 (periarticular histiocytic sarcoma) and Figure 2 (systemic histiocytic sarcoma).

### 3.8 Intrathoracic lymph node involvement

**Figure 3:**
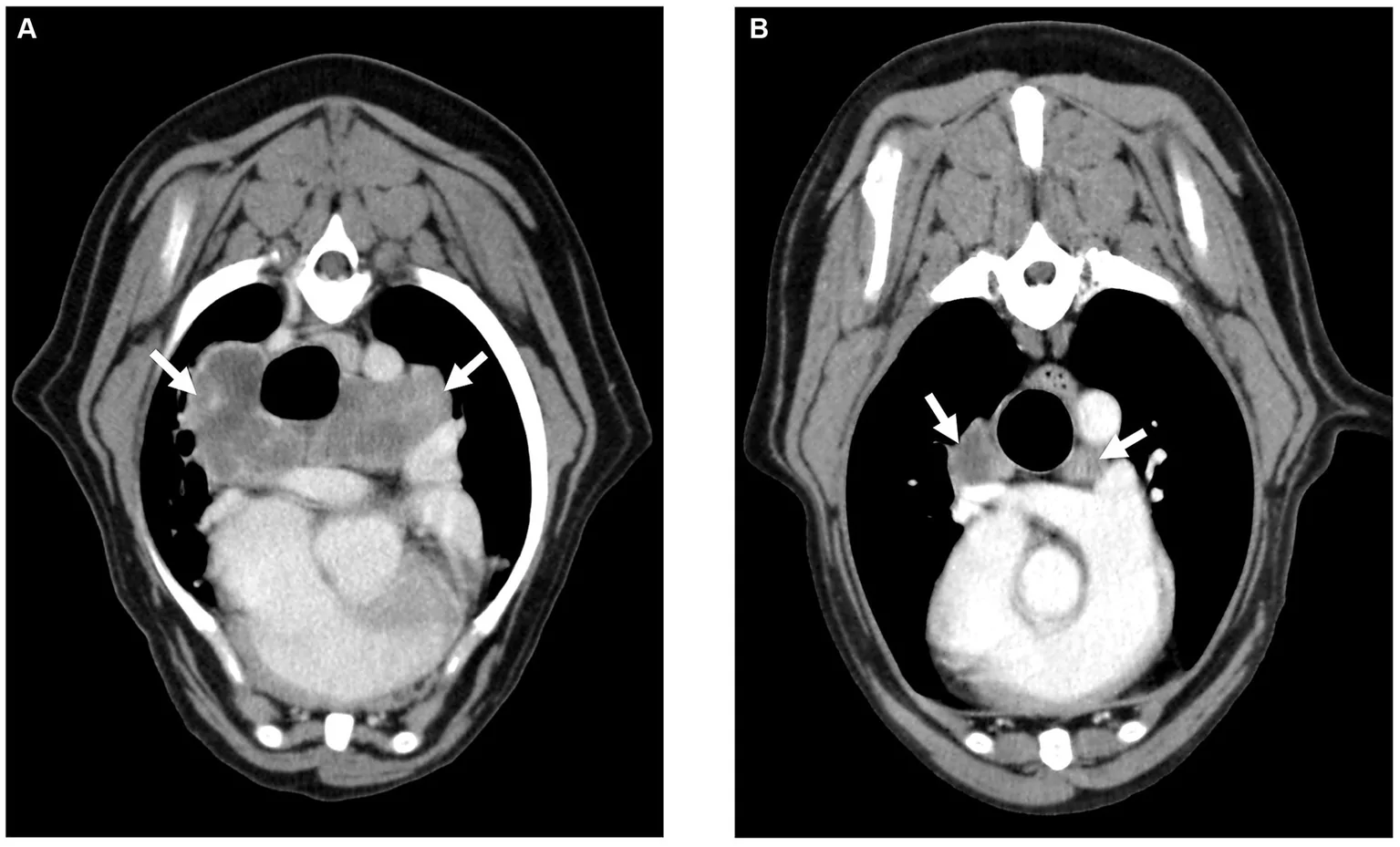
Transverse post contrast CT images of dogs displayed with a soft tissue reconstruction algorithm (WL = 50, WW = 350). The patient’s right is to the left of the image. **(A)** Bernese Mountain Dog (BMD), 6-year-old, male: Cross-sectional view at the level of the tracheobronchial lymph nodes of a dog with systemic histiocytic sarcoma. The severely enlarged and heterogeneous attenuating tracheobronchial lymph nodes are marked with white arrows. **(B)** Golden Retriever, 11-year-old, castrated male, primary lesion—right stifle: Cross-sectional view at the level of the tracheobronchial lymph nodes of a dog with periarticular histiocytic sarcoma. The tracheobronchial lymph nodes (arrows) are mildly enlarged and show a heterogeneous contrast enhancement.

The intrathoracic lymph nodes (tracheobronchial, cranial mediastinal, and sternal) were assessed for enlargement and volume (Figure 3). The lymph nodes in systemic HS patients tended to be more frequently enlarged. Twenty-one out of 26 (81%) showed enlarged lymph nodes compared to PAHS patients, where 12/19 (63%), displayed enlarged lymph nodes. In systemic HS, the lymph nodes were generally larger (19.4 cm^3^ ± 22.9 cm^3^) compared to PAHS patients (7.1 cm^3^ ± 10.1 cm^3^). However, these findings did not reach the level of significance (enlargement: *p* = 0.306, volume: *p* = 0.203).

## 4 Discussion

The results of this study demonstrate that PAHS differ significantly from systemic HS in terms of pulmonary lesion size, shape, intraparenchymal location, and distribution (Table 1). These findings indicate that PAHS and systemic HS are associated with distinct patterns of pulmonary involvement, which may reflect differences in biological behavior, metastatic spread, and raise the possibility of different biological subtypes within dendritic cell tumors.

**Table 1:** Computed tomography features of systemic and periarticular HS: Comparison of the intrathoracic findings in systemic HS and periarticular HS.

Feature	Systemic HS	PAHS	Statistics
Median number of pulmonary lesions	3 (IQR: 1–3; range 1–18)	4 (IQR: 1.5–7; range 1 to >50)	*p* = 0.181
Median lesion volume	103.7 cm^3^ (IQR: 33.1–155 cm^3^)	2.5 cm^3^ (IQR: 0.5–7.5 cm^3^)	*p* < 0.001^*^
Lesion shape	20/26 (77%) irregular	15/19 (79%) round to ovoid	*p* < 0.001^*^
Distribution pattern	20/26 (77%) ventral	11/19 (58%) generalized	*p* < 0.001^*^
Location within the parenchyma	23/26 (88%) bronchocentric	12/19 (63%) interstitial	*p* < 0.001^*^
Contrast enhancement	29.5 ± 12 HU	32 ± 13.2 HU	*p* = 0.461
Air bronchogram	Present in 26/26 (100%) of cases	Present in 7/19 (37%) of cases	*p* < 0.001^*^
Intrathoracic lymphadenopathy	21/26 (81%), mean volume 19.4 cm^3^ ± 22.9 cm^3^	12/19 (63%), mean volume 7.1 cm^3^ ± 10.1 cm^3^	*p* = 0.306 *p* = 0.203

*Statistically significant values.

Although localized and disseminated HS exhibit similar morphology and immunohistochemical profiles, the periarticular form shows unique characteristics that set it apart. Histiocytic sarcomas originate from interstitial dendritic cells (20), but the precise dendritic cell sublineage involved in canine HS remains to be determined (15).

Dogs with the periarticular form of HS tend to have a more favorable prognosis than those with HS in other anatomical locations (3). This outcome is observed regardless of metastatic status, although the presence of metastatic disease in dogs with PAHS appears to be a negative prognostic factor (3). In addition, recent studies have reported prolonged survival times in other localized forms of canine HS treated with surgery +/− chemotherapy, compared with systemic disease. In dogs with localized splenic HS, survival times exceeding 1 year have been described, which are comparable to those reported for dogs with primary pulmonary HS following surgical intervention and chemotherapy (13, 21). These findings suggest that localized HS may be associated with a more favorable clinical outcome when treated with aggressive multimodal treatment. Molecular data have also identified biological differences among HS subtypes. *PTPN11* mutations have been identified in canine HS and are associated with aggressive behavior and visceral dissemination, proposed to appear during later stages of tumor progression (22, 23). In addition, gene expression analyses in Flat-Coated Retrievers have demonstrated distinct differences between localized soft-tissue and visceral HS, supporting the presence of divergent biological mechanisms among HS subtypes (24).

Differences in lesion size and number between systemic HS and PAHS may reflect differences in tumor development. Twenty out of 26 (77%) patients with systemic HS presented with three or fewer lesions. Those lesions showed high tumor volumes, often involving a large proportion of the affected lobes, with a median volume of 103.7 cm^3^ (IQR: 33.1–155 cm^3^). In contrast, 11/19 (58%) of the patients diagnosed with PAHS exhibited more than three smaller lesions with a median volume of 2.5 cm^3^ (IQR: 0.5–7.5 cm^3^). Those differences in lesion size and number may be related to the fact that pulmonary involvement in systemic HS most likely represents a primary or early site of tumor involvement rather than metastatic spread. This may contribute to the development of fewer but larger and more confluent pulmonary lesions. In patients with the periarticular form, the primary tumor develops in periarticular tissues of large appendicular joints, with pulmonary lesions likely representing metastatic dissemination. This could explain why a higher number and smaller nodules were observed depending on the extent of the spread.

Additionally, the distribution of pulmonary lesions varied significantly between the two groups. In systemic HS, lesions were distributed mainly ventrally in 20/26 (77%) patients and were more likely located in the right middle lung lobe, which was involved in 16/26 (62%) patients, a finding consistent with earlier reports (13, 25–27). In contrast lesions in PAHS were distributed more evenly, correlating with lung lobe volumes. This difference in lesion distribution may reflect variations in the mode of spread within the body or the biological behavior.

The distribution observed in the systemic HS, particularly the ventral location and the frequent involvement of the right middle and left cranial lung lobes (26), which are also among the most commonly affected by aspiration pneumonia (28), may suggest a potential association with pulmonary regions predisposed to local inflammatory processes. Considering that the risk of developing PAHS increases with pre-existing joint disease and chronic inflammation (16), previous pulmonary inflammation may represent a potential contributing factor. However, the present study did not evaluate previous aspiration events or evidence of chronic pulmonary inflammation. Therefore, this hypothesis remains speculative and requires further investigations. The observed increased risk of PAHS in Bernese Mountain Dogs with orthopedic disease, together with a decreased risk in Bernese Mountain Dogs receiving long-term anti-inflammatory and joint-support treatments, supports the assumption that there is an association between local chronic inflammation and PAHS occurrence (29).

The lesions in patients diagnosed with the systemic HS form often exhibited a bronchocentric location, seen in 23/26 (88%) of the cases, and were irregular/confluent in shape in 20/26 (77%). Bronchocentric located lesions were present in all patients with systemic HS. In three patients, additional interstitial lesions were observed. Those lesions were smaller and may represent early metastasis of the primary lesions. In contrast, the pulmonary lesions in patients with PAHS were primarily interstitial in 12/19 (63%) and appeared round to ovoid in shape in 15/19 (79%) patients, which resembles the metastatic distribution in other high-grade sarcomas.

Remarkably, air bronchograms were present in at least one lesion in all cases of patients with systemic HS, whereas they were observed in only 7/19 (37%) of PAHS cases. One possible explanation could be that systemic HS may originate directly from dendritic cells in the lung parenchyma, which are enriched in peribronchial regions and could explain the bronchocentric growth pattern, whereas PAHS lesions more likely develop via hematogenous spread, resulting in randomly distributed interstitial nodules.

The features of the intrathoracic lesions observed in the systemic HS-group are consistent with previously described characteristics of pulmonary HS. Previous reports, including one study of 39 dogs with HS imaged with either plain radiography alone or computed tomography and another including 21 dogs with HS all imaged with radiography, described similar pulmonary findings (25, 26). In these two studies the BMD was the most affected breed. This aligns with our study, where systemic HS affected this breed most. Furthermore, the other breeds identified in our study align with the already known breed predispositions for this disease (11–13). The notably high incidence of HS in these breeds indicates an underlying genetic predisposition (9). In addition a recent study investigating the different features of pulmonary metastatic nodules reported that air bronchograms, larger nodule size (>1 cm) and thoracic lymphadenomegaly were associated with disseminated histiocytic sarcoma (30). Although that study did not differentiate between HS subtypes, these features were observed more often in the systemic HS group in the present study.

The pulmonary lesions in all cases showed mild to moderate contrast enhancement, which aligns with previously reported imaging findings in HS, where mild to moderate contrast enhancement was typically observed in intrathoracic histiocytic sarcoma lesions (25). No significant differences were noted in contrast enhancement between the systemic and the periarticular group.

Although a trend was observed in the number of lesions and the presence of intrathoracic lymphadenopathy, these findings did not achieve statistical significance, likely due to the relatively small number of patients in our study. However, intrathoracic lymphadenopathy was more frequent in systemic HS with 21/26 (81%) dogs, compared to PAHS with 12/19 (63%) dogs. This trend toward greater lymph node involvement in systemic HS is consistent with the disseminated nature of this neoplasia, which often involves multiple organ systems. As reported in previous studies, lymphadenopathy has been a frequent finding (16). Additionally, the intrathoracic lymph nodes tended to be larger in systemic HS patients than PAHS patients.

Despite these insights, certain limitations should be acknowledged, including the relatively small number of patients and the retrospective design that may have influenced the statistical significance of some findings. Furthermore, histopathologic examination of the pulmonary lesions was not performed in all patients, limiting definitive characterization of the observed lung lesions. Due to the lack of direct cytological or histopathological confirmation of the presumed metastatic pulmonary lesions, especially in the PAHS group, misclassification of some pulmonary lesions cannot be completely excluded. However, this was considered less likely due to the absence of evidence of concurrent disease and the presence of the confirmed histiocytic sarcoma with regional lymph node involvement indicating metastatic progression.

Furthermore, some degree of diagnostic heterogeneity within the systemic HS group cannot be completely excluded, as reliable immunohistochemical markers are lacking, making precise subtyping challenging. Therefore, the inclusion of cases with a primary pulmonary origin and secondary dissemination cannot be entirely excluded.

In addition, pulmonary lesion volume was estimated using an ellipsoid formula, which provides an approximation rather than an exact measurement of lesion volume and may have resulted in measurement errors that differed between the groups because of differences in lesion morphology.

Another limitation is that the observers were aware of the diagnosis at the time of image review, which may have introduced a potential source of observer bias. The images were evaluated for predefined features to ensure a consistent assessment.

Finally, CT examinations were performed using different scanner models. However, all examinations were performed using a 16-row CT scanner from Toshiba, with standardized patient positioning, contrast media application and reconstruction algorithms. Therefore, although technical differences may have existed, their impact on qualitative assessment was considered limited.

The distinct CT characteristics observed between systemic HS and PAHS indicate that these two forms of HS are associated with different patterns of pulmonary involvement despite the mentioned limitations. The findings of this study highlight that systemic HS presents with fewer, larger, irregularly shaped, bronchocentric, and mainly ventrally located lesions, often with air bronchograms. In contrast, PAHS is characterized by a greater number of smaller, interstitial and round-to-ovoid lesions distributed throughout the lungs with less frequent air bronchograms. The nodular lung pattern in PAHS resembles metastatic distribution in other high-grade sarcomas. In addition, intrathoracic lymph node enlargement was more frequently observed in systemic HS, and lymph nodes tended to be larger than in PAHS, although these differences were not statistically significant.

The significant differences in morphology and distribution may reflect differences in metastatic pathways and biological behavior between PAHS and systemic HS. These results align with previous studies that highlighted considerable variability in the morphological features of HS across different breeds, supporting the notion of distinct subtypes of HS, which may arise from different dendritic cell lineages or follow various differentiation pathways. Whether these imaging differences reflect distinct biological subtypes of canine HS remains to be determined and should be investigated in future histopathological, molecular, and genetic studies.

## Data Availability

The data supporting the findings of this study are available from the corresponding author upon reasonable request. Requests to access these datasets should be directed to MF, m.folie@tierklinik-hofheim.de.
